# Commentary: Transcranial Magnetic Stimulation over Left Inferior Frontal and Posterior Temporal Cortex Disrupts Gesture-Speech Integration

**DOI:** 10.3389/fnhum.2018.00256

**Published:** 2018-06-20

**Authors:** Linda Drijvers, James P. Trujillo

**Affiliations:** ^1^Centre for Language Studies, Radboud University, Nijmegen, Netherlands; ^2^Donders Institute for Brain, Cognition, and Behaviour, Radboud University, Nijmegen, Netherlands; ^3^Max Planck Institute for Psycholinguistics, International Max Planck Research School for Language Sciences, Nijmegen, Netherlands

**Keywords:** speech, gesture, transcranial magnetic stimulation (TMS), semantic integration, speech-gesture integration

Listeners integrate speech with gestures in face-to-face communication. This semantic integration of auditory and visual information is thought to occur automatically (Kelly et al., 2010). Previous neuroimaging studies have found inconsistent results on the involvement and role of the left inferior frontal gyrus (LIFG, e.g., Willems et al., 2007, 2009) and posterior middle temporal gyrus (pMTG, e.g., Holle et al., 2008, 2010; Straube et al., 2011) in this process. For example, Willems et al. (2009) suggested that pMTG is mostly involved in the integration of pantomimes (re-enactments of actions that can be understood without speech) and speech, whereas LIFG is more involved in the integration of iconic gestures (gestures that are ambiguous without speech) and speech. Extending on this, Holle et al. (2010) suggested that pSTS/MTG is involved in the integration of iconic gestures and speech to perform an initial conceptual matching between the inputs, whereas LIFG might be involved in processes of modulation and revision. Others have argued that LIFG/pMTG are both involved in combining the semantic information from both inputs (Dick et al., 2014)

A recent study in *The Journal of Neuroscience* by Zhao et al. (2018) investigated whether LIFG/pMTG are causally involved in speech-gesture integration by perturbing activity in these regions using continuous theta burst stimulation (cTBS) (Experiment 1) and repetitive transcranial magnetic stimulation (rTMS) (Experiment 2) relative to a control region. Participants were presented with videos of an actor/actress uttering action verbs while producing a gesture. In these videos, gender congruency and semantic congruency were manipulated. Participants were asked to identify the gender of the speaker after every video. In Experiment 1, Zhao et al. (2018) used cTBS to investigate whether disrupting activity in LIFG/pMTG would lead to a reduction of the semantic congruency effect. A disruption of brain activity was expected to lead to less distraction or competition by a mismatching gesture when a brain area was causally involved in speech-gesture integration. In Experiment 1, a reduction of the semantic congruency effect was indeed found for LIFG, but not pMTG. In Experiment 2, Zhao et al. (2018) did find a reduction of the semantic congruency effect in pMTG, but LIFG was not stimulated. The authors conclude that LIFG and pMTG likely work together during speech-gesture integration, and postulate that LIFG regulates strategic semantic access on temporal storage areas, whereas pMTG is involved in accessing supramodal representations.

We suggest that these results could be explained by the coupling between pMTG and LIFG. As the authors state that the LIFG and pMTG are tightly anatomically coupled, it is possible that stimulating one area would also result in (an unquantified) stimulation of the other (Silvanto and Pascual-Leone, 2008). An alternative explanation for the non-significant effect of stimulating the pMTG in Experiment 1 is that only the LIFG is causally involved in the integration of speech and higher-level visual semantic information, whereas pMTG is integrating low-level information from the auditory and visual modality. In this case pMTG stimulation leads to distal suppression of the LIFG, leading to a weakening of the semantic congruency effect. The effect is then less pronounced due to stimulation of the causal region (LIFG) being indirect. While Zhao et al acknowledge that non-stimulated brain areas might also be affected, this is an important limiting factor for using TMS for functional localization, in particular without quantifying indirect stimulation effects (Kim et al., 2016). In order to rule out this possibility, neuroimaging could be used to assess not only activation in the targeted region, but also of the non-targeted region. This would clarify whether LIFG was unaffected in Experiment 2, and further elucidate the separate roles for pMTG and LIFG in speech-gesture integration.

We therefore argue that the current study has demonstrated that TMS over LIFG/MTG disrupted the semantic integration of speech and gestures, but that these results do not demonstrate the functional roles of these areas during speech-gesture integration. For example, Zhao et al. (2018) argue that the potential role of LIFG might be the strategic recovery of context-appropriate semantic information. However, we believe that the observed reduction of the semantic congruency effect after stimulating LIFG in Experiment 1 could equally well be explained as a disruption of the unification of the gesture and speech signal, which might be more taxing when semantic incongruency increases integration load. This explanation fits with the MUC model (Hagoort, 2013). We therefore argue that future work could consider a different behavioral task to probe speech-gesture integration that does not include incongruent gestures, as these gestures could cause additional integration processes that might not be natural in everyday multimodal communication.

An interesting follow-up would be to use a different behavioral task and neuroimaging, combined with a measure of directional connectivity, such as dynamic causal modeling (DCM) (Friston et al., 2003), to truly unravel the functional roles of LIFG/pMTG. Directional connectivity analysis could quantify how LIFG and MTG interact during speech gesture integration without any stimulation, and subsequently determine how the system is affected by stimulation over LIFG, MTG, or both. As DCM allows one to model how stimuli and task manipulations affect specific model parameters (i.e., activity within regions, and coupling strength between regions), combining DCM with TMS could be useful for making inferences regarding the specific roles of regions and their connections.

In conclusion, utilizing brain stimulation provides novel, interesting and relevant findings regarding the role of frontal and temporal regions in speech-gesture integration. However, we suggest that without additional measures such as neuroimaging, as well as an integration measure that does not manipulate semantic congruency, it is not possible to wholly disentangle the roles of the LIFG and MTG in terms of their causal involvement in speech-gesture integration. Overall, the findings of Zhao and colleagues highlight the importance of the LIFG and MTG in speech-gesture integration, and provide an important and exciting foundation for future work to further explore the contribution of the two areas.

## Author contributions

LD and JT discussed the original idea and wrote the manuscript together.

### Conflict of interest statement

The authors declare that the research was conducted in the absence of any commercial or financial relationships that could be construed as a potential conflict of interest.
